# The Role of Amide Proton Transfer (APT)-Weighted Imaging in Glioma: Assessment of Tumor Grading, Molecular Profile and Survival in Different Tumor Components

**DOI:** 10.3390/cancers16173014

**Published:** 2024-08-29

**Authors:** Gonçalo Borges de Almeida, Riccardo Pascuzzo, Francesca Mambrin, Domenico Aquino, Mattia Verri, Marco Moscatelli, Massimiliano Del Bene, Francesco DiMeco, Antonio Silvani, Bianca Pollo, Marina Grisoli, Fabio Martino Doniselli

**Affiliations:** 1 Department of Neuroradiology, Hospital de São José, 1150-199 Lisbon, Portugal; 2 Neuroradiology Unit, Fondazione IRCCS Istituto Neurologico Carlo Besta, Via Celoria 11, 20133 Milan, Italy; 3 Neuroradiology Unit, Department of Diagnostics and Pathology, Azienda Ospedaliera Universitaria Integrata Verona, Piazzale Aristide Stefani 1, 37126 Verona, Italy; 4 Neurosurgery Unit, Fondazione IRCCS Istituto Neurologico Carlo Besta, Via Celoria 11, 20133 Milan, Italy; 5 Department of Oncology and Hematology-Oncology, Università Degli Studi di Milano, 20122 Milan, Italy; 6 Department of Neurological Surgery, Johns Hopkins Medical School, Baltimore, MD 21205, USA; 7 Neuro-Oncology Unit, Fondazione IRCCS Istituto Neurologico Carlo Besta, Via Celoria 11, 20133 Milan, Italy; 8 Neuropathology Unit, Fondazione IRCCS Istituto Neurologico Carlo Besta, Via Celoria 11, 20133 Milan, Italy

**Keywords:** glioma, glioblastoma, IDH, MGMT, survival, MRI, chemical exchange saturation transfer

## Abstract

**Simple Summary:**

In this retrospective study of 61 patients with diffuse gliomas, APT-weighted imaging in the solid tumor component provided quantitative metrics associated with WHO grade and survival. Mean APT values of the necrotic component showed high variability between subjects, warranting further investigation. These results highlight the importance of assessing the different tumor components with APT-weighted imaging, to provide useful diagnostic and prognostic information for the management of patients with gliomas.

**Abstract:**

Amide Proton Transfer-weighted (APTw) imaging is a molecular MRI technique used to quantify protein concentrations in gliomas, which have heterogeneous components with varying cellularity and metabolic activity. This study aimed to assess the correlation between the component-specific APT signal of the neoplasm and WHO grade, molecular profile and survival status. Sixty-one patients with adult-type diffuse gliomas were retrospectively analyzed. APT values were semi-automatically extracted from tumor solid and, whenever present, necrotic components. APT values were compared between groups stratified by WHO grade, IDH-mutation, MGMT promoter methylation and 1- and 2-year survival status using Wilcoxon rank-sum test, adjusting for multiple comparisons. Overall survival (OS) was analyzed in the subgroup of 48 patients with grade 4 tumors using Cox proportional-hazards models. Random-effects models were used to assess inter-subject heterogeneity of the mean APT values in each tumor component. APT values of the solid component significantly differed between patients with grades 2–3 and 4 tumors (mean 1.58 ± 0.50 vs. 2.04 ± 0.56, *p* = 0.028) and correlated with OS after 1 year (1.81 ± 0.58 in survivors vs. 2.17 ± 0.51 in deceased patients, *p* = 0.030). APT values did not differ by IDH-mutation, MGMT methylation, and 2-year survival status. Within grade 4 glioma patients, higher APT kurtosis of the solid component was a negative prognostic factor (hazard ratio = 1.60, *p* = 0.040). Mean APT values of the necrosis showed high inter-subject variability, although most necrotic tumors were grade 4 and IDH wildtype. In conclusion, APTw imaging in the solid component provided metrics associated with glioma grade and survival status but showed weak correlation with IDH-mutation and MGMT promoter methylation status, in contrast to previous works. Further research is needed to understand APT signal variability within the necrotic component of high-grade gliomas.

## 1. Introduction

Amide Proton Transfer-weighted (APTw) imaging is a Magnetic Resonance (MR) contrast-agent-free molecular imaging technique which generates signal based on endogenous cellular mobile proteins and peptides [1]. The underlying principle of APTw imaging is chemical exchange-dependent saturation transfer (CEST) [2,3], by which amide protons in proteins may be selectively saturated and exchanged with water protons. The origin of APT signal in tumors is not fully understood, but it may be linked to higher mobile protein concentrations in malignant cells and increased cellularity; APT signal can also be affected by the local pH and temperature through alterations in the proton exchange rate [3]. Therefore, in brain tumors, the presence of areas of great APT values variability are commonly seen.

Since cellular proteins and peptides are known to be highly expressed in rapidly growing high-grade gliomas [4], this imaging technique may be helpful in differentiating high-grade from low-grade gliomas, often in addition to contrast-enhanced sequences [5], proton MR spectroscopy [6], diffusion-weighted imaging and perfusion-weighted imaging [7]. Compared to these current MRI techniques, APTw imaging alone may even show higher sensitivity and specificity in assessing tumor grade [8] and may show strong correlation with tumor proliferation (Ki67 proliferative index) [6]. Likewise, APTw imaging has also been able to predict overall survival and progression-free survival in gliomas [9,10]. Although there is a paucity of descriptive studies with extensive case series of different pathologies, a growing corpus of data pertaining to the various histotypes of brain tumors and their manifestation in APT is gradually being published in the literature [11].

The 2016 [12] and 2021 [13] World Health Organization (WHO) classifications of brain tumors have underlined the importance of newly discovered molecular markers which influence tumor behavior and therefore the disease course. Isocitrate dehydrogenase (IDH) mutations in gliomas have been shown to be associated with a better clinical prognosis and overall survival compared to IDH-wildtype tumors [14,15]. APTw imaging has been shown to be able to predict IDH-mutation status in patients with both low-grade [16] and high-grade [10] gliomas, also when APT-derived radiomic features were used [17]. Another gene which has been shown to carry prognostic value in gliomas is O6-methylguanine-DNA methyltransferase (MGMT). There is currently very limited evidence supporting the role of APTw imaging in predicting MGMT promoter methylation status. To the best of our knowledge, there are a few studies comparing methylated and unmethylated tumors using APTw imaging [10,18,19], but only one was able to reach statistical significance [18]. Overall, the evidence supporting the use of APTw imaging in predicting the genetic profile of gliomas is scarce.

The purpose of this study is to measure the APT signal in different locations of adult-type diffuse gliomas and assess its correlation with glioma grading, molecular profile (IDH-mutation and MGMT promoter methylation status), and survival.

## 2. Materials and Methods

### 2.1. Study Design

This study was approved by the institutional Ethics Committee (SEURAT study, approved on 17 May 2023).

Our database was screened between May 2020 and May 2023. Patients who underwent preoperative MRI for suspected glioma at the *Fondazione IRCCS Istituto Neurologico Carlo Besta* in Milan, Italy, were consecutively included if they matched the following criteria: (a) patients undergoing preoperative imaging study at high-field MRI (3T); (b) patients for whom the APT sequence was included in the diagnostic protocol of the preoperative MRI study; (c) patients who underwent surgery at our institution with histologically proven diffuse glioma (astrocytoma, oligodendroglioma, or glioblastoma classified following the WHO 2021 CNS tumor classification [13]) of any grade. Patients in whom the APT sequence was affected by motion artifacts were excluded.

For all patients, the update on prognosis was obtained from the institutional database (“Cancer Registry”), which is based on the Italian “*Istituto Nazionale di Statistica*” (ISTAT) database. The current update is as of 5 May 2024.

### 2.2. Molecular Analysis

#### 2.2.1. MGMT

The methylation pattern of the CpG islands of the MGMT gene was determined by chemical transformation of unmethylated cytosines to uracil, followed by methylation-specific PCR using primers specific for both methylated DNA and modified, unmethylated DNA [20]. Treatment of tumor DNA (1 μg) with sodium bisulfate was performed with the CpG Genome DNA Modification kit (Intergen, Purchase, NY, USA), following the protocol provided by the manufacturer. Universally methylated DNA (Intergen) was used as a positive control, while DNA obtained from normal lymphocytes was used as a negative control. A methylation-specific PCR assay was conducted with fluorescently labeled primers. The methylation-specific PCR products (1.5 μL) were transferred to 8% polyacrylamide gels, examined with an automatic DNA sequencer (Alf Express II, Amersham Biosciences Roosendaal, The Netherlands), and quantified with the Alf Win Fragment Analyzer program (version 1.02, Amersham Biosciences). The ratio between the peak of the PCR products deriving from methylated or unmethylated DNA of the same neoplasm was calculated; values higher than 0.1 were interpreted as presence of MGMT promoter methylation.

#### 2.2.2. IDH

DNA was extracted from freshly collected and frozen samples using a *BioRobot EZ1* (Qiagen, Hilden, Germany). The *3730 DNA Analyzer* (Applied ByoSystems, Waltham, MA, USA) performed Sanger sequencing of the p.Arg132 region of IDH1 (codons 55–138) and the p.Arg172 region of IDH2 (codons 151–179). An immunohistochemical assay was performed on formalin-fixed tumor brain tissue samples with the antibody clone H09 (Dianova, Eching, Germany), capable of reacting specifically at the R132H mutation point of IDH1 (1:10 dilution for 30 min, at room temperature). Standard deparaffinization, rehydration and heat-induced epitope retrieval (HIER) procedures were applied. Using a second conjugated antibody, an indirect immunoenzymatic labeling was performed with detection system based on biotin/streptavidin.

### 2.3. MRI Acquisition

MRI studies were acquired with a 3T MRI scanner (3T Philips Achieva Dstream) using the following study protocol:

- 3D T1-weighted sequence after administration of contrast medium (Gadovist, gadobutrol 1 mmol/mL, Bayer AG^®^, Leverkusen, Germany) fast-field-echo (FFE): Repetition time/echo time (TR/TE) = 9.93 ms/4.5 ms; flip angle (FA) = 8°; slice thickness = 1 mm; no gap = 1 mm; matrix = 240 × 240 mm; field of view (FOV) = 240 × 240 mm;
- 3D FLAIR sequence: TR/TE = 4800 ms/333 ms; TI = 1650 ms; slice thickness = 1 mm; no gaps; matrix = 240 × 240 mm; FOV = 240 × 240 mm;
- APT: 3D TSE DIXON sequence, saturation radiofrequency (RF) pulse duration 2.0 s; B1 power 2.0 µT; 40 sync Gaussian pulses each of 50 ms; 7 off-resonance saturation pulses: ±3.1, ±3.5, ±3.9 and −1560 ppm (S0); 9 slices; FOV 212 × 183 × 40 mm; matrix = 116 × 116 reconstructed 224 × 224; voxel size 1.8 × 1.8 × 4.4 mm; TR = 3825 ms; TE = 6.2 ms; FA = 90°; refocusing angle 120°; Echo train length (ETL) 181; Spectral presaturation with inversion recovery (SPIR) fat suppression. Total acquisition time was 4 min and 30 s.

### 2.4. Image Procesing and Analysis

**Figure 1:**
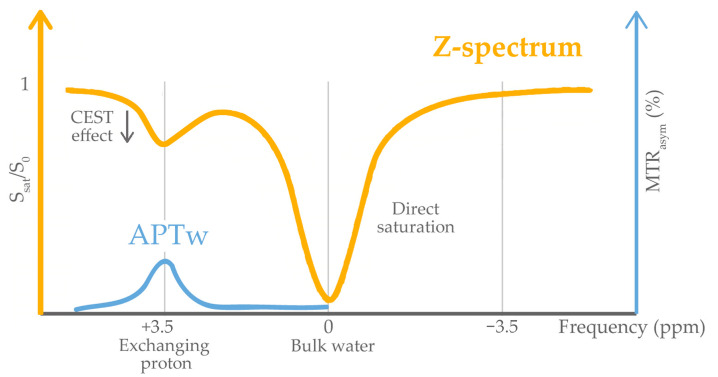
The Z-spectrum (orange) represents the water signal saturation (S_sat_/S_0_) as a function of the frequency offset of the RF saturation pulse. In the presence of amide groups, there is a signal drop at +3.5 ppm. Furthermore, there is full signal saturation at 0 ppm (bulk water), because the RF saturation pulse directly saturates the proton spins in the water molecules. From the Z-spectrum, the so-called magnetization transfer asymmetry (MTR_asym_) (blue) is assessed and measured in percent (%). For APTw imaging, MTRasym is calculated as the difference between the Z-spectrum at −3.5 ppm and at +3.5 ppm, normalized to the S_0_ image (measured without RF saturation). This specific MTRasym is being referred to as APTw. Modified from [21].

Post-processing of APT images was automatically performed by the Philips 3T MRI system after image acquisition, as mentioned in the Philips 3D APT whitepaper [21]. APTw imaging is usually quantified in terms of a magnetization transfer ratio asymmetry (MTR_asym_) analysis with respect to the water frequency (0 ppm in the Z-spectrum) at an offset of ±3.5 ppm [22]. A full Z-spectrum fitted for all off-resonance frequencies was obtained for each voxel to correct B_0_ nonuniformities. This Z-spectrum was aligned per voxel and centered at the point of maximum direct water saturation (0 ppm). Then, MTR_asym_ at ±3.5 ppm was calculated using the formula:

MTR_asym_(+3.5 ppm) = [S_sat_(−3.5 ppm) − S_sat_(+3.5 ppm)]/S_0_, (1)

where S_0_ and S_sat_ refer to MRI signal without and with saturation RF pulse, respectively. Finally, the resulting MTR_asym_ value at 3.5 ppm was displayed as percent level (relative to S_0_) in the APTw images (Figure 1).

Two neuroradiology residents (G.B.A. and F.M.) designed volumetric regions of interest (ROIs) on APT images in APT space using the ITK-SNAP software [23] (version 3.8.0). A first ROI was placed in correspondence with the solid component of the tumor (named “lesion” ROI), excluding necrosis, large cysts, hemorrhage or large vessels with the aid of the 3D contrast-enhanced T1 and 3D FLAIR images acquired during the same MRI examination in which the APT was also acquired. An additional circular ROI (named “necrosis” ROI) with a fixed diameter of 10 pixels was drawn in correspondence with the area of tumor necrosis, whenever present, avoiding hemorrhagic areas. Another ROI was also drawn on the normal white matter contralateral to the lesion (normal-appearing white matter, “NAWM”) as control. A neuroradiologist with 8 years of experience (F.M.D.) checked the segmentations and corrected the ROIs if they had been drawn too close to the skull or bony structures of the skull base.

From each ROI, the following statistical parameters of APT values were extracted using MATLAB 2022b (MathWorks, Natick, MA, USA): mean, median, standard deviation, 10th percentile, 90th percentile, skewness and kurtosis.

### 2.5. Statistical Analysis

For each ROI, a random-effects model was used to pool mean APT values of the patients, assuming that there was not only one true mean APT value common to all patients, but a distribution of true mean APT values [24]. A random-effects model allows to estimate the parameters of the distribution of the mean APT values, considering the presence of possible between-patient heterogeneity. Specifically, the restricted maximum likelihood estimator [25] was used to calculate the variance τ^2^ of the true mean APT value underlying the data. We additionally performed Cochran’s Q test of heterogeneity [26] to verify whether the variability in the observed mean APT values was larger than would be expected based on sampling variability alone; consequently, we computed the I^2^ statistic [27] defined as the percentage of variability in the mean APT values that was caused by between-subject heterogeneity. We used Knapp–Hartung adjustments [28] to calculate the confidence interval around the pooled mean APT. When substantial between-patient heterogeneity was present, 95% prediction intervals were also calculated [29] to illustrate which range of true mean APT values could be expected in other (future) patients.

APT statistical parameters were compared between groups of patients stratified by WHO grade (2–3 vs. 4), IDH-mutation status, MGMT promoter methylation status, 1- and 2-year survival using Wilcoxon rank-sum test, adjusting for multiple comparisons by using Benjamini–Hochberg false-discovery-rate correction. Overall survival analysis was conducted using univariate and, when multiple factors were significant, multivariate Cox proportional-hazards models.

Statistical significance was set at *p* < 0.05. Statistical analyses were performed in R (version 4.1.2); “metafor” package was used for fitting random-effects models and “survival” package was used for the survival analysis.

## 3. Results

Our retrospective search found 74 patients, of which 61 patients were included with pathology-proven gliomas (7 were excluded for other diagnosis such as lymphoma or glioneuronal tumor, 6 for motion artifacts). Demographics and tumor characteristics are reported in Table 1. The majority of patients were male (74%) and presented with WHO grade 4 (79%), IDH-wildtype (75%) and MGMT-methylated (58%) tumors. Most patients were alive (64%) one year after the diagnosis, while few were still alive (33%) after 2 years.

### 3.1. Between-Subject Variability of Mean APT Values in Tumor Compartments and Other Tissues

Mean APT values in the “lesion” ROI ranged approximately between 1 and 3 (Supplementary Figure S1). The pooled mean APT value of this ROI across subjects was estimated at 1.74 (95% CI: 1.60–1.87) by the random-effects model. The between-subject heterogeneity variance was estimated at τ^2^ = 0, with an I^2^ value of 0%, indicating no significant variability among subjects (Cochran’s Q test of heterogeneity: Q_(d_._f_. _= 60)_ = 41.89, *p* = 0.9636).

**Figure 2:**
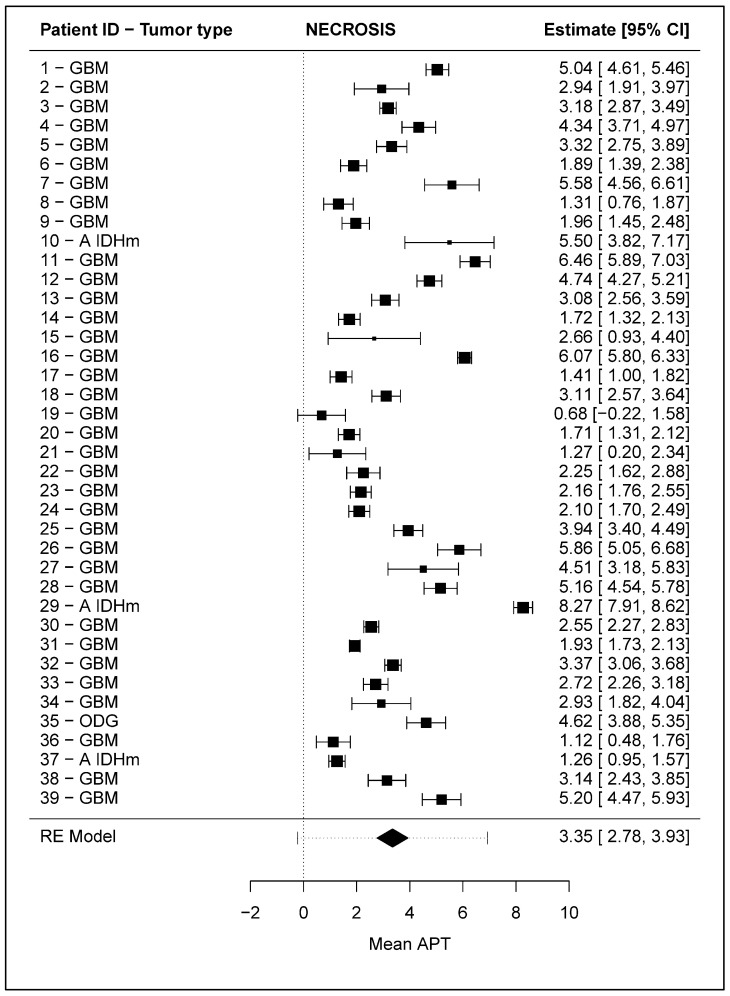
Forest plot representing the mean APT values in the “necrosis” ROI of the 39 included patients. Horizonal bars represent the 95% confidence interval of the mean APT values. On the right, numerical values of mean APT (“Estimate”) and 95% confidence interval (“[95% CI]”) are reported. At the bottom, the estimate of the average APT mean values across patients, obtained through a random-effects model (“RE Model”) based on restricted maximum likelihood, is reported graphically as a diamond and numerically indicated on the right column. A prediction interval, represented as a dashed horizontal bar for the RE-Model estimate, is also illustrated for the pooled mean APT in order to give a range into which we can expect the mean APT values of other patients to fall based on present evidence. GBM: glioblastoma; A IDHm: astrocytoma IDH mutant; ODG: oligodendroglioma.

Brain MRI showed necrotic areas in 39 patients. Most of their mean APT values ranged between 1 and 6 (Figure 2). The pooled mean APT value of this ROI across subjects was estimated at 3.35 (95% CI: 2.78–3.94) by the random-effects model. The between-subject heterogeneity variance was estimated at τ^2^ = 3.04, with an I^2^ value of 98%, indicating significantly high variability among subjects (Cochran’s Q test of heterogeneity: Q_(d_._f_. _= 38)_ = 2406.25, *p* < 0.0001). Since between-subject heterogeneity was high, a 95% prediction interval for the mean APT value in the necrosis was also computed (range: 0–7).

**Figure 3:**
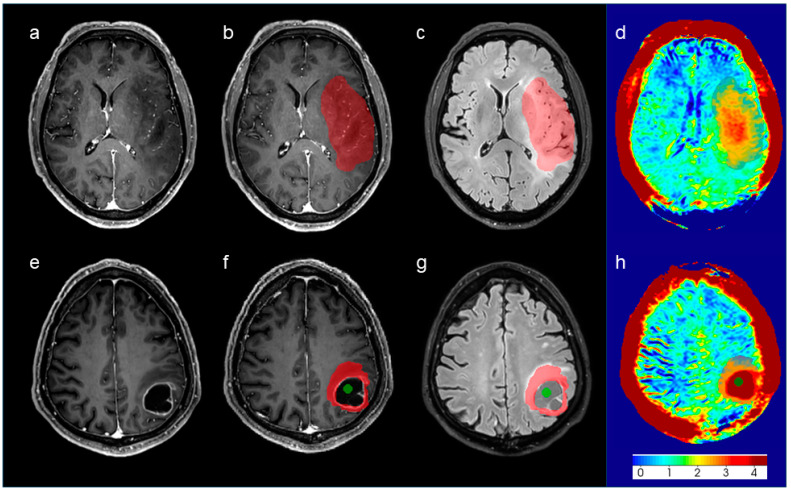
Diffuse astrocytoma grade 2, IDH-mutant, (**a**–**d**) and glioblastoma, IDH-wildtype (**e**–**h**), are shown. Images in the first two columns (**a**,**b**,**e**,**f**) and in the third column (**c**,**g**) represent post-gadolinium volumetric T1 sequences and FLAIR volumetric images, respectively. Images in the fourth column (**d**,**h**) show the corresponding color-coded APT signal. Both patients presented with space-occupying lesions within the left cerebral hemisphere, the second showing necrotic components. Red ROI represents the solid component (“lesion ROI”), while green ROI represents the necrotic component (“necrosis ROI”).

Figure 3 exemplifies this disparity of APT values between two patients with glioma, with and without necrosis and of different WHO grades.

Mean APT values in the “normal appearing white matter” (NAWM) ROI ranged mostly between 0 and 1 (Supplementary Figure S2). The pooled mean APT value of this ROI across subjects was estimated at 0.36 (95% CI 0.31–0.41) by the random-effects model. The between-subject heterogeneity variance was estimated at τ^2^ = 0, with an I^2^ value of 0%, indicating no significant variability among subjects (Cochran’s Q test of heterogeneity: Q_(d_._f_. _= 60)_ = 48.7, *p* = 0.8514).

### 3.2. Correlation of APT-Derived Statistical Parameters with Tumor and Patient Characteristics

#### 3.2.1. “Lesion” ROI

Within the “lesion” ROI, several APT parameters were correlated with WHO grade, IDH mutation status, MGMT methylation status and survival status 1 and 2 years after the diagnosis. Statistically significant values were obtained when comparing APT parameters with tumor grading and 1-year survival status (Table 2 and Table 3 and Supplementary Table S1), as patients with WHO grade 4 tumors and deceased patients after 1 year showed higher APT values than patients with lower grades gliomas and patients still alive after 1 year, respectively. No statistically significant differences were obtained regarding the molecular profile of gliomas, although patients with IDH-wildtype tumors tended to have higher APT values. The APT values of gliomas with unmethylated and methylated MGMT promoter were almost identical. Overall survival and 2-year survival analyses did not show any significant difference, although APT values tended to be higher in patients who died after 2 years than in those who were still alive.

A survival analysis was conducted in the subgroup of 48 patients with WHO grade 4 tumors, to investigate the relationship between APT values and survival rate in a more homogeneous group. We found that a solid tumor component with lower APT kurtosis values and undergoing a gross total resection were positive prognostic factors according to both univariate and multivariate models (Table 4).

#### 3.2.2. “Necrosis” ROI

Among the 39 patients with necrosis, the vast majority were WHO grade 4 gliomas (37 out of 39, 95%), while only two patients had WHO grade 3 gliomas. As for IDH-mutation status, the vast majority were IDH-wildtype gliomas (35 out of 39, 90%), while only four had IDH-mutant gliomas. Instead, MGMT methylation and survival status after 1 and 2 years were both more balanced: MGMT promoter methylation was found in 19 out of 37 (51%) patients with available data, 21 out of 37 patients were alive after 1 year, 9 out of 28 patients were alive after 2 years. For these reasons, APT statistical parameters were compared only between groups stratified by MGMT promoter methylation and survival statuses and not by WHO grade nor by IDH-mutation status. APT values were not statistically different concerning the MGMT promoter methylation status and between alive and deceased patients both after 1 and 2 years. Nonetheless, patients with MGMT-methylated tumors and deceased patients presented slightly higher APT values (Table 5). Overall survival analysis did not show any significant result (Table 6).

Focusing on the 22 patients who did not have necrosis, 11 had WHO grade 4 and 11 had WHO grade 2–3 gliomas. Grouping the patients according to the IDH-mutation status produced the same subdivision as based on WHO grade (i.e., 11 IDH-wildtype and 11 IDH-mutant gliomas, respectively). The MGMT promoter was methylated in 15 patients and unmethylated in the remaining 7. After 1 year, 18 patients were alive and 4 died; among those patients who had a follow-up of at least 2 years, 10 were alive and 10 died. There were no statistically significant differences concerning the APT values between patients grouped by their WHO tumor grading, molecular profile, survival status after 1 and 2 years, nor for the overall survival analysis (Supplementary Tables S2 and S3).

## 4. Discussion

In this study, the APT signal of pathology-proven gliomas was measured in both solid (“lesion” ROI) and necrotic (“necrosis” ROI) components of gliomas, evaluating their inter-subject variability and their correlation with tumor grading, molecular profile and survival status. We found no significant variability concerning the solid component and the NAWM, indicating a high degree of APT signal repeatability among patients. Our results are in line with a previous study showing high repeatability of APT signal in the supratentorial brain in healthy patients, as well as in patients with glioma and stroke-affected regions [30]. A recently published study also demonstrated reproducible APT measurements across scan sessions and scanners [31], which is essential to draw reliable conclusions and compare studies performed at different centers.

We found that grade 2–3 gliomas had statistically significantly lower APT values than WHO grade 4 gliomas in the solid tumor component. Several studies attempted to assess the correlation between APT values and WHO tumor grading [32,33,34,35,36,37,38,39,40,41]. The majority of these studies were able to stratify gliomas according to their WHO grade based on APT, with pooled results showing that APTw signal intensity increases with increasing histological grade [42], in line with our findings. As APT values may partially overlap between low-grade and high-grade gliomas, adding the 90th percentile APT value has been shown to be a reliable parameter as it may pick the “hot spot” region seen in high-grade tumors [7].

We also found a significant correlation with survival status after 1 year in the solid component of the tumor, with higher APT values associated with shorter survival time. These results are in line with previous studies which found a negative correlation between APT values and overall survival [9,10], as APT values reflect the aggressiveness and proliferative index of gliomas [6]. However, a correlation between 2-year survival and APT values was not observed, indicating that APT may be able to capture some key tumor characteristics with an impact on shorter-term survival, as tumors at advanced developmental stages tended to show high APT values.

When focusing on patients with WHO grade 4, we found that low APT kurtosis values in the solid tumor component correlated with better prognosis, regardless of the type of surgical resection (complete or partial), which is a known prognostic factor [43]. Kurtosis is a measure of the tailedness of a distribution, associated with the frequency with which very high or very low values relative to the mean are present in the sampled tissue (ROI). A possible explanation for this correlation can be found in the heterogeneity of tissue with high APT kurtosis, possibly more infiltrated by neoplasia; a tissue with lower APT kurtosis, more homogeneous in the distribution of APT values, could be characterized by less infiltration of healthy tissue and thus better prognosis.

Our study failed to show a correlation of APT values with the molecular profiles of gliomas. Nonetheless, we found slightly higher values in the solid tumor component in patients with IDH-wildtype gliomas than IDH-mutant gliomas, which has been previously demonstrated in both histologically proven low-grade [16] and high-grade [10,17] gliomas. The absence of significant differences in our study may be possibly due to the relatively low number of WHO grade 2 glioma patients or the different tumor classification, based on the WHO 2021 classification. Moreover, our statistical approach included correction for multiple comparisons, which was not always performed in previous studies. Protein expression in mutant IDH1 glioma cells is downregulated compared to IDH1-wildtype cells, which may account for the lower APT signal seen in IDH-mutant gliomas [44]. In the last decade, several target therapies for IDH-mutant gliomas have been developed [45], although no molecularly targeted therapies have currently been approved. The noninvasive determination of IDH-mutation status using APTw imaging could potentially allow patients to benefit from targeted therapy before surgery.

We also failed to demonstrate relevant differences in APT signal according to the MGMT promoter methylation status. Considering the solid component, we found almost equivalent APT values in most statistical parameters between MGMT-methylated and -unmethylated tumors. These findings are in contrast with previous studies reporting lower APT values in MGMT-methylated tumors [10,18,19]. We hypothesize that this difference may be due to our relatively small sample size. Preoperative determination of MGMT promoter methylation status is important to optimize treatment strategies for patients with gliomas. As patients with unmethylated MGMT tumors may be unresponsive to temozolomide, several alternative treatment modalities have been proposed for these patients which include fractionated radiotherapy, chemotherapy agents independent of MGMT repair, immunotherapy and tumor-treating fields [46]. The role of APTw imaging in detecting MGMT promoter methylation should be further investigated in future studies.

To the best of our knowledge, no previous studies investigated APT values in the necrotic component of gliomas. We could not find any significant difference regarding survival nor MGMT promoter methylation status, although MGMT-methylated tumors presented slightly but not significantly higher APT values.

In fact, APT values derived from the necrotic tumor area displayed high variability among subjects, despite the homogeneity of the population with a necrotic component (WHO grade 4 (95%), IDH-wildtype (90%) tumors). Several factors may influence the APT signal in tumors other than protein concentration, such as the type of necrosis (liquefactive or coagulative). Liquefactive necrosis is characterized by the presence of a liquid substance that facilitates high protein mobility, resulting in a high APT signal. On the other hand, coagulative necrosis is characterized by the formation of a gelatinous substance and therefore impairs free protein mobility, which results in low APT signal [47]. A similar mechanism has been demonstrated in an experiment performed with eggs, in which the solid-like cooked albumin displayed lower APT signal than liquid albumin because of the decreased protein mobility [48]. On MRI, liquefactive necrosis has high apparent diffusion coefficient (ADC) values [49] due to free water and protein motion, whereas coagulative necrosis shows restricted diffusion, especially in high-grade tumors treated with bevacizumab [49] or radiotherapy [50]. Unfortunately, in our study, APT signal could not be correlated with the type of necrosis as this was not mentioned in the neurosurgical reports nor routinely analyzed by neuropathologist. To our knowledge, only one study has correlated the type of necrosis with overall survival in patients with hepatocellular carcinoma [51], reporting lower overall survival in patients with liquefactive necrosis. Since both types of necrosis may be seen in glioblastomas [49,52], further research is needed to assess whether the type of necrosis can be correlated with overall survival.

There are some limitations to this study. First, the monocentric and retrospective design of our study does not permit to test the reproducibility of our results. Multicentric and prospective studies with standardized acquisition parameters and image processing are therefore warranted. Second, even though most of our patients underwent gross total resection, some were diagnosed after brain biopsy. Therefore, the possibility of undersampling of tissue cannot be completely excluded due to the heterogeneity of the tumor. Third, the low number of WHO grade 2 and grade 3 gliomas did not permit to differentiate lower grades in two separate classes. Fourth, we used MTR_asym_ at +3.5 ppm as the metric to obtain APTw values, which may be confounded by the nuclear Overhauser enhancement (NOE) effects from non-exchangeable protons resonating near to water protons [53]. Nonetheless, there is evidence that APT effect is still the major contributor to APTw image contrast when using MTR asymmetry analysis [53]. More recently, alternative metrics and improved acquisition approaches have been developed to obtain more reliable CEST effects and disentangle APT signal from relaxation shine-through effects [54].

## 5. Conclusions

Our study demonstrated significant correlations of APT values in the solid component of the tumor with WHO grading and survival status. In particular, APT kurtosis of the solid component could be considered as a biomarker for measuring tumor infiltration, and, if confirmed, it could be useful for prognosis assessment. Although we failed to achieve a statistically significant association with IDH-mutation and MGMT methylation status, we still found minor differences in APT values according to the molecular profile of gliomas, which influences treatment choices. Interestingly, we also found quite variable APT values within the necrotic components, which warrants further research and histopathological correlation to assess its clinical value.

## Figures and Tables

**Table 1:** Demographics and tumor data of the 61 included patients.

Characteristic	Value
Age, median (range) years	56 (23–76)
Sex (M/F)	45/16
WHO grade (2/3/4)	3/10/48
Tumor volume, median (range) mm^3^	30, 187 (67–167, 636)
IDH mutation (Yes/No)	15/46
MGMT promoter methylation (Yes/No) ^1^	34/25
Survival status after 1 year (Alive/Dead)	39/22
Survival status after 2 years (Alive/Dead) ^2^	19/38

^1^ MGMT promoter methylation status was not available in two patients. ^2^ Survival status after 2 years was not available in four patients at the time of analysis. Data are number of patients unless otherwise specified. M: male; F: female; WHO: World Health Organization; IDH: isocitrate dehydrogenase; MGMT: O6-Methylguanine-DNA-methyltransferase.

**Table 2:** APT values of the tumor solid component stratified by group.

APT Parameter	WHO Grade		IDH Status		MGMT Promoter Status	
	2–3 (*n* = 13)	4 (*n* = 48)	Mutant (*n* = 15)	Wildtype (*n* = 46)	Methylated (*n* = 34)	Unmethylated (*n* = 25)
Mean	1.58 * (0.50)	2.04 * (0.56)	1.69 (0.55)	2.02 (0.57)	1.91 (0.53)	1.93 (0.63)
Median	1.55 * (0.52)	1.99 * (0.57)	1.65 (0.54)	1.98 (0.58)	1.85 (0.53)	1.90 (0.64)
10th percentile	0.74 * (0.29)	1.08 * (0.42)	0.79 (0.31)	1.08 (0.43)	0.99 (0.39)	1.01 (0.45)
90th percentile	2.41 * (0.72)	3.07 * (0.84)	2.64 (0.89)	3.03 (0.83)	2.91 (0.84)	2.88 (0.87)
Skewness	0.35 (0.46)	0.27 (0.46)	0.41 (0.49)	0.25 (0.44)	0.37 (0.48)	0.20 (0.43)
Kurtosis	3.45 (1.17)	3.03 (0.63)	3.43 (1.16)	3.01 (0.60)	3.21 (0.90)	3.03 (0.61)

Data are average and, in parenthesis, standard deviation of several statistical parameters extracted from the APT map in the “lesion” region of interest, grouped according to WHO grade, IDH mutation status, and MGMT promoter methylation status. Asterisks indicate statistically significant differences with an adjusted *p*-value < 0.05 using a Benjamini–Hochberg procedure to control the false discovery rate, as reported in Supplementary Table S1. APT: amide proton transfer; WHO: World Health Organization; IDH: isocitrate dehydrogenase; MGMT: O6-Methylguanine-DNA-methyltransferase.

**Table 3:** Correlation between APT values of the tumor solid component and survival.

APT Parameter	Survival Status after 1 Year		Survival Status after 2 Years		Overall Survival	
	Alive (*n* = 39)	Dead (*n* = 22)	Alive (*n* = 19)	Dead (*n* = 38)	HR (95% CI)	*p*-Value
Mean	1.81 * (0.58)	2.17 * (0.51)	1.78 (0.61)	2.01 (0.54)	1.55 (0.95–2.54)	0.081
Median	1.76 * (0.58)	2.13 * (0.51)	1.74 (0.63)	1.96 (0.54)	1.55 (0.94–2.54)	0.085
10th percentile	0.93 (0.44)	1.15 (0.34)	0.89 (0.42)	1.04 (0.38)	1.54 (0.76–3.11)	0.227
90th percentile	2.74 * (0.84)	3.27 * (0.79)	2.73 (0.92)	3.04 (0.79)	1.33 (0.98–1.82)	0.070
Skewness	0.31 (0.42)	0.25 (0.53)	0.31 (0.43)	0.26 (0.48)	0.90 (0.44–1.84)	0.768
Kurtosis	3.02 (0.84)	3.29 (0.66)	3.10 (1.05)	3.14 (0.66)	1.13 (0.83–1.53)	0.444

Data in the first four columns are average and, in parenthesis, standard deviation of several statistical parameters extracted from the APT map in the “lesion” region of interest, grouped according to survival status after 1 and 2 years. The last two columns report the hazard ratios of the APT parameters with 95% confidence intervals, along with the corresponding *p*-values, computed with univariate Cox proportional-hazards models. Asterisks indicate statistically significant differences with an adjusted *p*-value < 0.05 using a Benjamini–Hochberg procedure to control the false discovery rate, as reported in Supplementary Table S1. APT: amide proton transfer; WHO: World Health Organization; HR: hazard ratio; CI: confidence interval.

**Table 4:** Survival analysis in the subgroup of patients with WHO grade 4 glioma.

Factor		Overall Survival (Univariate)		Overall Survival (Multivariate)	
		HR (95% CI)	*p*-Value	HR (95% CI)	*p*-Value
APT Mean		0.86 (0.51–1.46)	0.576	-	-
APT Median		0.89 (0.53–1.48)	0.640	-	-
APT 10th percentile		0.71 (0.35–1.43)	0.335	-	-
APT 90th percentile		0.94 (0.66–1.33)	0.709	-	-
APT Skewness		0.99 (0.46–2.11)	0.977	-	-
APT Kurtosis		1.66 (1.07–2.56)	0.023 *	1.60 (1.02–2.52)	0.040 *
Surgery type	Biopsy (*n* = 11)	ref	-	ref	-
	GTR (*n* = 23)	0.31 (0.13–0.72)	0.006 *	0.29 (0.12–0.68)	0.005 *
	Partial (*n* = 13)	0.90 (0.39–2.08)	0.810	0.76 (0.32–1.81)	0.536
Age at surgery		1.02 (0.99–1.05)	0.256	-	-

Hazard ratios of the APT parameters in the solid tumor component and other factors are reported with 95% confidence intervals, along with the corresponding *p*-values, computed with univariate Cox proportional-hazards models. Factors with *p* < 0.10 at univariate analysis were considered in the same multivariate Cox proportional-hazards model to evaluate the effect of each significant factor adjusted for the presence of the other significant variables. Asterisks indicate significant differences with *p* < 0.05. APT: amide proton transfer; HR: hazard ratio; CI: confidence interval; GTR: gross total resection; ref: reference level.

**Table 5:** APT values of the tumor necrotic component stratified by group.

APT Parameter	MGMT Promoter Status		Survival Status after 1 Year		Survival Status after 2 Years	
	Methylated (*n* = 19)	Unmethylated (*n* = 18)	Alive (*n* = 21)	Dead (*n* = 18)	Alive (*n* = 9)	Dead (*n* = 28)
Mean	3.69 (1.89)	3.13 (1.67)	3.05 (1.79)	3.72 (1.73)	3.63 (2.38)	3.28 (1.56)
Median	3.72 (1.92)	3.13 (1.69)	3.06 (1.81)	3.74 (1.77)	3.64 (2.39)	3.29 (1.59)
10th percentile	3.20 (1.85)	2.75 (1.65)	2.70 (1.79)	3.19 (1.70)	3.30 (2.38)	2.81 (1.51)
90th percentile	4.15 (1.97)	3.51 (1.67)	3.41 (1.81)	4.22 (1.74)	3.95 (2.36)	3.75 (1.61)
Skewness	−0.24 (0.54)	0.01 (0.42)	−0.10 (0.47)	−0.12 (0.52)	−0.08 (0.53)	−0.12 (0.50)
Kurtosis	2.55 (0.62)	2.40 (0.75)	2.48 (0.71)	2.41 (0.65)	2.41 (0.57)	2.51 (0.71)

Data are average and standard deviations of several statistical parameters extracted from the APT map in the “necrosis” region of interest, grouped according to MGMT promoter methylation status and survival status after 1 and 2 years. No statistically significant differences were found after adjusting *p*-values using a Benjamini–Hochberg procedure to control the false discovery rate. APT: amide proton transfer; MGMT: O6-Methylguanine-DNA-methyltransferase.

**Table 6:** Survival analysis in the subgroup of patients presenting with a tumor necrotic component.

APT Parameter	Overall Survival	
	HR (95% CI)	*p*-Value
Mean	0.98 (0.81–1.19)	0.828
Median	0.98 (0.81–1.18)	0.823
10th percentile	0.95 (0.77–1.16)	0.600
90th percentile	1.01 (0.84–1.21)	0.932
Skewness	0.90 (0.44–1.83)	0.768
Kurtosis	1.14 (0.71–1.83)	0.578

Hazard ratios of the APT parameters are reported with 95% confidence intervals, along with the corresponding *p*-values, computed with univariate Cox proportional-hazards models. APT: amide proton transfer; HR: hazard ratio; CI: confidence interval.

## Data Availability

The original data presented in the study are available in the institutional repository of the Fondazione IRCCS Istituto Neurologico Carlo Besta (https://doi.org/10.5281/zenodo.13385707 (accessed on 20 August 2024)).

## Supplementary Materials

The following supporting information can be downloaded at: https://www.mdpi.com/article/10.3390/cancers16173014/s1, Figure S1: Forest plot representing the mean APT values in the “lesion” ROI of the 61 included patients; Figure S2: Forest plot representing the mean APT values in the “normal appearing white matter” ROI of the 61 included patients; Table S1: Adjusted *p*-values (with Benjamini–Hochberg procedure) of the comparisons reported in Table 2 and Table 3; Table S2: APT values of the tumor solid component in the subgroup of 22 patients without necrosis component, stratified by group: Table S3: Overall survival analysis in the subgroup of 22 patients without necrosis component.
