# Supplementary material for: The Role of Amide Proton Transfer (APT)-Weighted Imaging in Glioma: Assessment of Tumor Grading, Molecular Profile and Survival in Different Tumor Components
Source: Cancers (Basel). 2024 Aug 29;16(17):3014. doi: 10.3390/cancers16173014 (PMC11394364; doi:10.3390/cancers16173014)
Supplement: Supplementary file 1 [file cancers-16-03014-s001.zip › cancers-3128702-supplementary.pdf]

Supplementary Materials

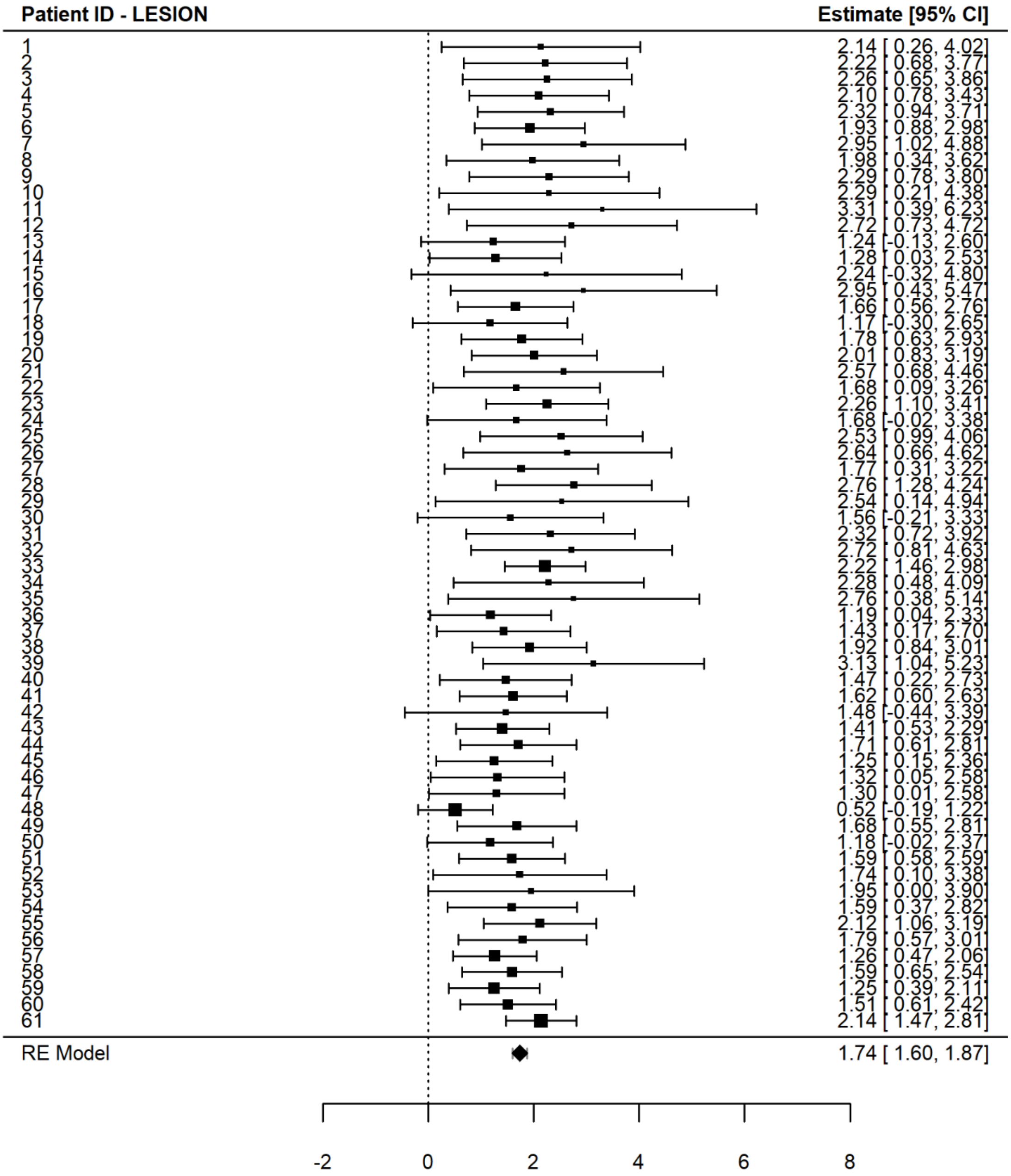

Figure S1. Forest plot representing the mean APT values in the "lesion" ROI of the 61 included patients. Horizonal bars represent the 95% confidence interval of the mean APT values. On the right, corresponding numerical values of mean APT ("Estimate") and 95% confidence interval ("[95% CI]") are reported. At the bottom, the estimate of the average mean APT values across patients, obtained through a random-effect model ("RE Model") based on restricted maximum likelihood, is reported graphically and numerically.

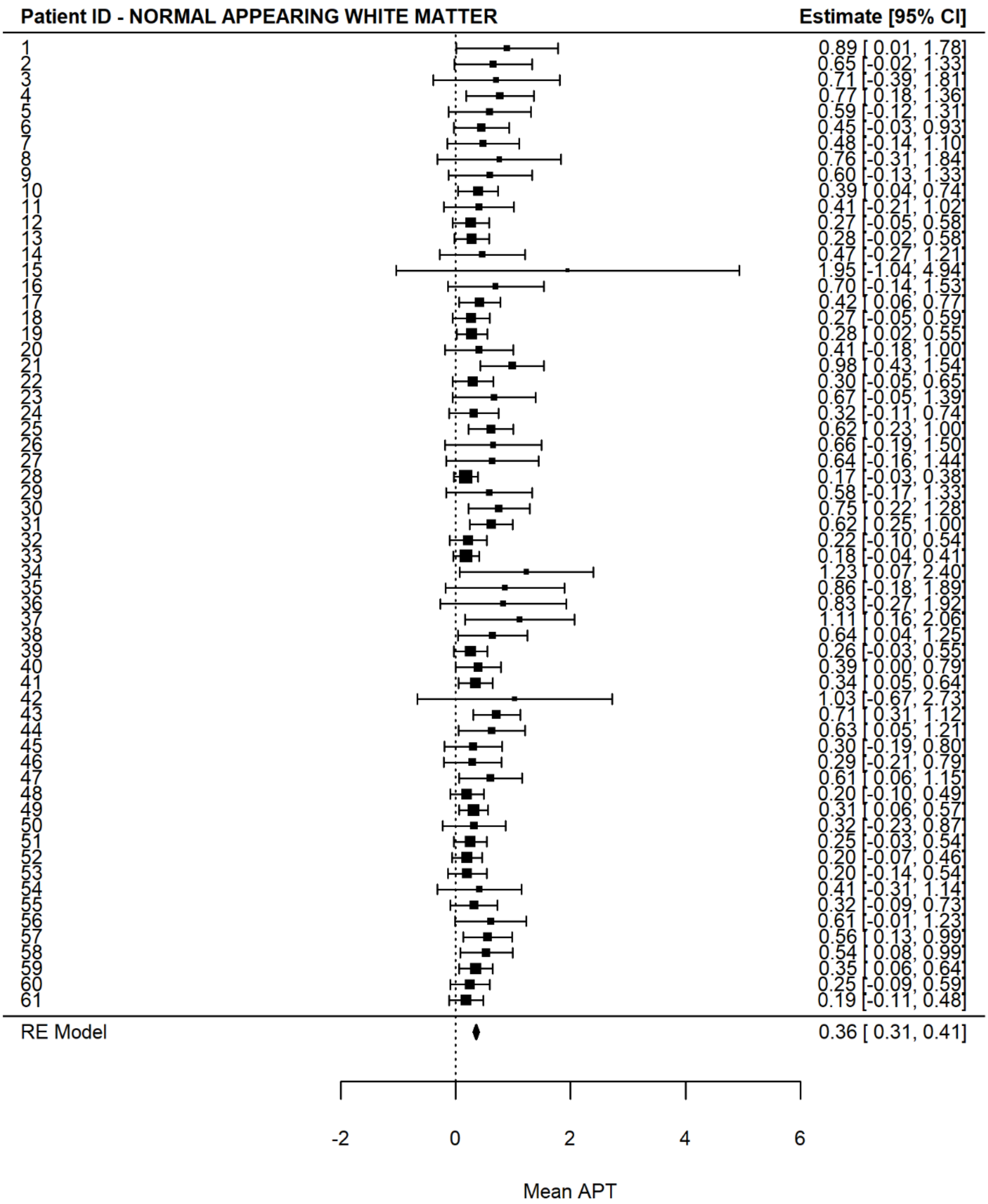

Figure S2. Forest plot representing the mean APT values in the "normal appearing white matter" ROI of the 61 included patients. Horizonal bars represent the 95% confidence interval of the mean APT values. On the right, corresponding numerical values of mean APT ("Estimate") and 95% confidence interval ("[95% CI]") are reported. At the bottom, the estimate of the average mean APT values across patients, obtained through a random-effect model ("RE Model") based on restricted maximum likelihood, is reported graphically and numerically.

Table S1. **Adjusted p-values (with Benjamini-Hochberg procedure) of the comparisons reported in Table 2 and 3.**

APT parameter	WHO grade	IDH status	MGMT promoter status	Survival status after 1 year	Survival status after 2 year
Mean	0.0275	0.1731	0.8781	0.0302	0.3798
Median	0.0275	0.1731	0.8781	0.0302	0.3798
10th percentile	0.0275	0.1658	0.8781	0.0632	0.3798
90th percentile	0.0275	0.1731	0.8781	0.0302	0.3798
Skewness	0.8824	0.5560	0.8781	0.8174	0.9999
Kurtosis	0.3389	0.3583	0.8781	0.0602	0.6889

Abbreviations: APT = amide proton transfer; WHO = World Health Organization; IDH = isocitrate dehydrogenase; MGMT = O6-Methylguanine-DNA-methyltransferase.

Table S2. **APT values of the tumor solid component in the subgroup of 22 patients without necrosis component, stratified by group.**

APT parameter	WHO grade			Survival status after 1 year			Survival status after 2 years			MGMT promoter status		
	Lower grade (n=11)	High grade (n=11)	p	Alive (n=18)	Dead (n=4)	p	Alive (n=10)	Dead (n=10)	p	Meth. (n=15)	Non-meth. (n=7)	p
Mean	1.48 (0.38)	1.56 (0.33)	0.8470	1.52 (0.38)	1.51 (0.19)	0.9671	1.48 (0.40)	1.50 (0.29)	0.5288	1.62 (0.28)	1.31 (0.41)	0.1061
Median	1.46 (0.40)	1.53 (0.36)	1.0000	1.50 (0.40)	1.48 (0.28)	0.7743	1.45 (0.42)	1.48 (0.34)	0.6842	1.60 (0.30)	1.27 (0.45)	0.0777
10th percentile	0.70 (0.28)	0.86 (0.40)	0.7179	0.79 (0.37)	0.74 (0.25)	1.0000	0.69 (0.28)	0.77 (0.28)	0.8798	0.87 (0.36)	0.60 (0.26)	0.1049
90th percentile	2.27 (0.53)	2.29 (0.37)	0.8470	2.27 (0.46)	2.31 (0.46)	1.0000	2.29 (0.56)	2.26 (0.37)	0.5787	2.39 (0.36)	2.05 (0.55)	0.1417
Skewness	0.29 (0.48)	0.14 (0.49)	0.7477	0.23 (0.44)	0.15 (0.72)	0.9023	0.34 (0.48)	0.09 (0.48)	0.4359	0.19 (0.55)	0.28 (0.30)	0.4902
Kurtosis	3.30 (1.20)	2.87 (0.52)	0.1932	3.03 (0.99)	3.33 (0.64)	0.2622	3.32 (1.27)	2.91 (0.53)	0.3527	3.23 (1.05)	2.77 (0.56)	0.0659

Data are average and, in parenthesis, standard deviations of several statistical parameters extracted from the APT map in the “lesion” ROI in patients without necrosis component, grouped according to WHO grade (‘high’=4, ‘lower’=2-3), survival status after 1 and 2 years, and MGMT promoter methylation status. Of note, grouping by WHO grade or IDH mutation status in this subgroup was identical. The “p” columns report unadjusted p-values, and none of them were below the significance level of 0.05.

Table S3. Overall survival analysis in the subgroup of 22 patients without necrosis component.

**APT parameter**	**HR**	**95% CI**	**p-value**
Mean	0.80	0.16-4.04	0.7918
Median	0.85	0.18-3.94	0.8342
10th percentile	0.79	0.13-4.71	0.7983
90th percentile	0.88	0.23-3.31	0.8513
Skewness	0.40	0.07-2.14	0.2840
Kurtosis	0.76	0.30-1.93	0.5595

Abbreviations: APT = amide proton transfer; HR = hazard ratio; CI = confidence interval.
